# Welcome to *Membranes*—A New Open Access Journal on Membrane Separation and Technology

**DOI:** 10.3390/membranes1010001

**Published:** 2010-12-12

**Authors:** Spas D. Kolev

**Affiliations:** Founding Editor-in-Chief, School of Chemistry, The University of Melbourne, Victoria 3010, Australia; E-mail: s.kolev@unimelb.edu.au

Membrane separation, from its infancy as a predominantly laboratory technique in the middle of the last century, has advanced with remarkable speed and now underpins a range of mature industrial technologies.

Porous membranes play a crucial role in many commercial applications: the desalination of seawater and brackish water via reverse osmosis and nanofiltration; the clean-up of industrial effluents by micro- and ultra-filtration or electrodialysis; and the separation of multi-component gas mixtures, to name only a few. At the same time, non-porous membranes have been employed in the purification of organic and inorganic compounds by means of techniques such as pervaporation and liquid membrane separation. Membranes are also indispensable in non-industrial applications such as healthcare (e.g., hemodialysis) and chemical analysis (e.g., membrane introduction mass spectrometry and flow injection analysis with online separation by gas-diffusion or dialysis). Industrial membrane separation technologies are cost effective, energy efficient and environmentally friendly.

Modern membrane technologies have been built upon foundations established by rigorous fundamental and applied research. Thousands of researchers worldwide are involved in the development, study and application of membrane-based separation processes. The prodigious increase in publications in this area since the 1950s, when only a few journal articles appeared in the literature, to the several thousand articles published last year, is testimony to the growing importance of membrane applications in everyday life. Numerous articles describing aspects of membrane research are published daily, and there is therefore a need for platforms where original results and ideas can be presented and discussed with minimal delay. The new open access journal *Membranes* will provide such a platform and I feel privileged to introduce its first issue. Authors publishing in *Membranes* as an open access journal will benefit of the high availability and visibility of their publications. The Editorial Office of the Publisher will professionally handle all submissions through a stringent peer-review procedure and professional production, whilst minimizing the delay of publication.

*Membranes* will cover research in the broad areas of (i) membrane development and associated structural and chemical studies; and (ii) membrane separation processes and their environmental, industrial, clinical and analytical applications. The Editorial Board of *Membranes* and I invite you to submit your articles to this new journal.

